# Preliminary findings on the development of a predictive model for BLCA based on disulfidptosis-associated IncRNAs signature

**DOI:** 10.1186/s12894-024-01454-3

**Published:** 2024-03-26

**Authors:** Chao Tang, Yanpeng Fan, Shusheng Zhu

**Affiliations:** 1 Department of Urology, Affiliated Yantai Yuhuangding Hospital, Qingdao University, Yantai, Shandong China; 2 Department of Urology, The First Hospital of Jilin University, Changchun, Jilin China; 3 Department of Urology, Jining No. 1 People’s Hospital, Jining, 271000 shandong China

**Keywords:** Disulfidptosis gene, Bladder urothelial carcinoma, Immune therapy, Immune microenvironment infiltration

## Abstract

**Background:**

Bladder urothelial carcinoma (BLCA) is the most common malignancy of the urinary tract, presenting with a wide range of clinical symptoms and prognosis. Disulfidptosis is a newly identified cell death method and closely associated with BLCA progression, prognosis, and treatment outcome. Currently, we need to construct a new prognostic model for disulfidptosis-related long noncoding RNAs (drlncRNAs) to improve the treatment strategy of BLCA.

**Methods:**

The data for BLCA samples were obtained from The Cancer Genome Atlas (TCGA), and then 10 unique genes related to disulfidoptosis (DRGs) were identified from research papers. The differences between the two groups showed in this study were used to create the “disulfidptosis-related long noncoding RNAs score” (disulfidptosis-score) prognostic model.

**Results:**

We identified two groups of drlncRNAs with high and low disulfidptosis scores in this study. Patients with low disulfidptosis scores had a better overall survival rate compared to those with high scores in bladder cancer, and the high disulfidptosis score subtype exhibited more active malignant pathways related to cancer than the low score subtype. We found that the low disulfidptosis-score subgroup had better prognosis than the high disulfidptosis-score subgroup. The expression of mutation burden was much higher in the low disulfidptosis-score group than in the high disulfidptosis-score group. The low disulfidptosis-score subgroup of patients exhibited significantly higher proportions of plasma cells, T cells CD8, and Tregs, while the high-risk subgroup had a greater abundance of Macrophages M0 and Macrophages M2. The disulfidptosis-score showed a strong correlation with the sensitivity of chemotherapeutic drugs, and patients in the low disulfidptosis-score group were more likely to exhibit an immune response and respond positively to immunotherapy. Additionally, we developed a nomogram to enhance the accuracy of the disulfidptosis-clinical score.

**Conclusion:**

Based on our investigation of disulfidptosis-score in BLCA, disulfidptosis-score may have an important role in TME, prognosis, and drug sensitivity. We also investigated the significance of the disulfidoptosis-score in relation to immunotherapy and immune response, providing a basis for improving prognosis and responding to immunotherapy among patients with BLCA.

**Supplementary Information:**

The online version contains supplementary material available at 10.1186/s12894-024-01454-3.

## Introduction

Malignant tumor is a major public health problem which seriously endangers human health and social development [1]. Bladder urothelial carcinoma (BLCA) is the most common malignancy of the urinary system and is the fourth most common cancer in men [2, 3]. The highest incidence of bladder cancer currently falls in most developed regions of the world, with approximately 550,000 new cases each year [4]. BLCA is a heterogeneous disease, comprising several tumor subtypes with differences in histology, genomic aberrations, and prognosis [5]. The mode of cell death has an important role in the development of BLCA [6]. Therefore, the exploration of cell death methods may help to discover the underlying mechanisms of tumor development.

Disulfidptosis is a novel mode of cell death, which differs from apoptosis, ferroptosis, pyroptosis, cuproptosis, and necrosis [7, 8]. In the issue of Nature Cell Biology, Liu et al. [7] investigated that the susceptibility of the actin cytoskeleton to disulfide stress mediates disulfidptosis. Based on the research paper in PubMed, we selected 10 genes associated with disulfidptosis identified through basic experiments for modeling. SLC7A11 and SLC3A2 (which encodes an SLC7A11 chaperone) were identified as suppressor. SLC7A11^high^-induced cell death under glucose starvation is disulfidptosis [7]. RPN1 knockdown made UMRC6 cells more resistant to disulfidptosis [7]. NCKAP1 deletion attenuated disulfidptosis in UMRC6 cells [7]. Glycogen synthase (GYS1) and various genes involved in mitochondrial oxidative phosphorylation (such as NDUFS1, OXSM, NDUFA11, NUBPL and LRPPRC) were identified as synergistic effect (whose inactivation synergizes with glucose starvation to induce cell death) [7]. Tumorigenesis and progression are characterized by the interplay of multiple genes and signaling pathways. It is not enough to study only a few genetic biomarkers associated with BLCA prognosis. Therefore, we have developed a classification system for 10 genes related to disulfidptosis and a disulfidptosis-score model, which could offer valuable insights into predicting the prognosis of BLCA and guiding clinical decision-making.

The purpose of the present paper was to construct a predictive model for BLCA based on disulfidptosis-associated IncRNAs signature. In this study, we used transcriptome data and clinical information from 431 BLCA patients to construct a BLCA scoring model (disulfidptosis-score). Additionally, we identified nine disulfidptosis-related long noncoding RNAs (drlncRNAs). Subsequently, the patients were categorized into two groups of drlncRNAs and a disulfidptosis-score system was established. The clinical utility of this scoring model was confirmed in BLCA patients, encompassing prognosis, immune microenvironment, and drug sensitivity.

## Methods

### Collection of datasets and information about samples

The flowchart is showed in Supplementary Figure S1. The data on gene expression, gene mutation, and clinical information of BLCA samples were collected from The Cancer Genome Atlas (TCGA) database (https://tcga-data.nci.nih.gov/tcga/). This dataset includes 412 BLCA samples and 19 normal samples with detailed information for further analysis. The median follow-up was 534 days (quartile range: 401 to 641 days) and 230 deaths were reported. The data was downloaded and managed in accordance with TCGA guidelines.

### Selection and differential expression analysis of drlncRNAs

According to the research of Liu et al. [7], we selected 10 DRGs (GYS1, NDUFS1, OXSM, LRPPRC, NDUFA11, NUBPL, NCKAP1, RPN1, SLC3A2, SLC7A11) for analysis. Then, the correlation between DRGs and differentially expressed lncRNAs was analyzed. All 306 drlncRNAs met the standard of Pearson’s correlation coefficient (|PearsonR|) > 0.4 and *p* < 0.001.

### Development and validation of a prognostic model

LASSO-Cox analysis was performed using the “glmnet” R package to reduce the risk of over-fitting. Multivariable Cox analysis was employed to identify candidate genes for constructing a prognostic model (disulfidptosis-score). The disulfidptosis-score was calculated as follows:

$${\sum }_{i=1}^{n}\text{E}\text{x}\text{p} \left(\text{l}\text{n}\text{c}\text{R}\text{N}\text{A}\right) \times \text{c}\text{o}\text{e}\text{f} \left(\text{l}\text{n}\text{c}\text{R}\text{N}\text{A}\right)$$

Where coef (lncRNA) was the regression coefficient and Exp(lncRNA) was the expression level of drlncRNAs. The “survminer” package was utilized to determine the cutoff point. Based on the disulfidptosis-score, we used Kaplan-Meier analysis to visualize the survival curve for two cohorts. Statistical significance was defined as *p*-values less than 0.05.

### Gene set enrichment analysis

Gene set enrichment analysis is a popular framework for condensing information from gene expression profiles into a pathway or signature summary [9]. GSVA can provide increased power to detect subtle pathway activity changes over a sample population. This approach was conducted to elucidate the variances in biological processes between two groups with different disulfidptosis scores, utilizing the “GSVA” R packages. The gene sets from the MSigDB database, specifically “c2.cp.kegg.Hs.symbols.gmt”, were downloaded for further GSVA analysis. By applying the criteria of | log2(Fold Change) | > 1 and false discovery rate (FDR) < 0.05 [10], we utilized the R package limma to identify a list of differentially expressed genes (DEGs). DEGs were analyzed using clusterProfiler R package for GO and KEGG, with FDR cutoff < 0.05. The clinical characteristics, including age, gender, TNM stage, and grade were analyzed. Additionally, we utilized the survival package of R software to conduct Kaplan-Meier survival analysis on various groups.

### Correlation and stratification analyses of the disulfidptosis-score

Univariable and multivariable Cox regression analyses were conducted to determine whether the disulfidptosis-score is an independent prognostic marker, taking into account both risk score and clinical characteristics variables. For the analysis of gene mutations, we obtained information on genetic alterations from the TCGA database. The R package “Maftools” was utilized to analyze gene mutations in different risk groups. In addition, our study examined the correlation between disulfidptosis-score and total mutation burden (TMB).

### Identification of immune characteristics for the disulfidptosis-score

CIBERSORT (https://cibersort.stanford.edu/) is a fundamental algorithm for determining the cellular composition of solid tumors or gene expression profiles. In our study, we utilized this tool to analyze the enrichment of immune cells in relation to disulfidptosis-score.

### Assessment of immunotherapy and drug sensitivity

In order to assess the prognostic value of the disulfidptosis-score in predicting immunotherapy outcomes for BLCA patients, we conducted a time-dependent receiver operating characteristic (ROC) curve analysis to determine the area under the curve (AUC). The sensitivity of different drugs in BLCA patients was predicted based on their disulfidptosis-score groups. The oncoPredict R package was utilized for drug prediction [11]. The Wilcoxon signed-rank test was used to investigate the difference in IC50 values among different risk groups, and the results were visualized using the R package “ggplot2”.

### Nomogram construction

Based on the independent prognosis outcome, a predictive nomogram was generated using clinical characteristics and disulfidptosis-score with the rms R package. In the scoring system, each variable has a corresponding score, and the scores of all variables in each sample are summed to obtain the total score [12]. The ROC curves were used to assess the accuracy of the nomogram in predicting 1-, 3-, and 5-year survival rates, while the calibration plots of the nomogram were utilized to demonstrate expected survival outcomes for these time periods based on observed results.

### Statistical analysis

The data analysis was performed using R software (version 4.1.3; https://www.R-project.org) and R Bioconductor packages. Kaplan-Meier survival curves and log-rank analysis were utilized to evaluate the differences in survival time among groups. The independent prognostic value of the risk signature was validated through univariable and multivariable Cox regression analysis. The validity of the model was confirmed through analysis of the receiver operating characteristic (ROC) curve. According to the correlation between the disulfidptosis-score and patient survival, we determined the optimal cutoff point of survival information for each cohort using the Survminer package. The hazard ratio (HR) for regulators and genes related to disulfidptosis was calculated using the univariable Cox regression model. To determine whether the disulfidptosis-score could serve as an independent prognostic predictor, we conducted a multivariable Cox regression analysis incorporating both the disulfidptosis-score and relevant clinical parameters associated with disulfidptosis. The differences between groups were examined using both Student’s t-test, Chi-square test and Wilcoxon signed rank test, with a significance level of *p* < 0.05 for all analyses.

## Result

### Identification of drIncRNA

**Fig. 1:**
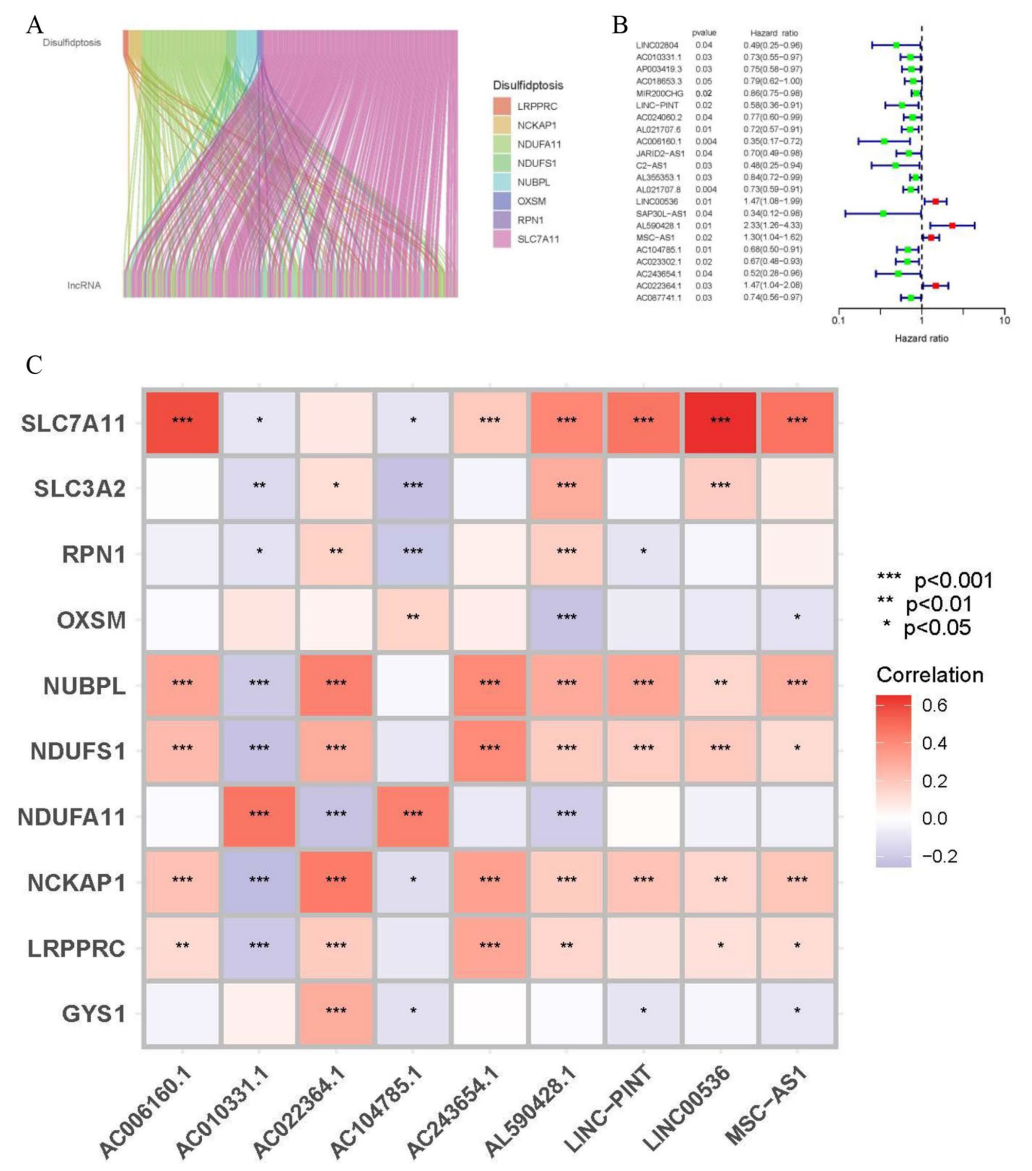
Identification of prognostic drlncRNAs in BLCA. (**A**) The Sankey diagram demonstrates correlation between disulfidptosis-related genes and drlncRNAs. (**B**) The prognostic drlncRNAs identified by uni-Cox regression analysis. (**C**) Correlations between drlncRNAs in the risk model and disulfidptosis-related genes. DrlncRNAs, disulfidptosis-related long noncoding RNAs.

We identified 306 lncRNAs that exhibited co-expression with disulfidptosis-related genes in BLCA and constructed a network graph to visualize the co-expression relationships between disulfidptosis-related genes and drlncRNAs (Fig. 1A). Through univariat Cox regression analysis, we identified a total of 22 drlncRNAs, comprising 4 high-risk drlncRNAs and 18 low-risk drlncRNAs (Fig. 1B). The lncRNAs with the most significant differential expression were chosen to create a correlation heat map (Fig. 1C).

### Prognostic model construction and evaluation

**Fig. 2:**
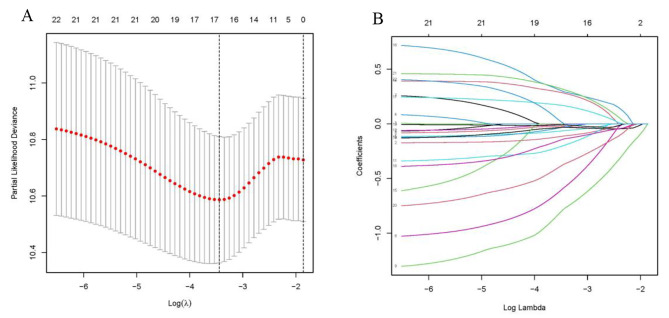
Extraction of drlncRNAs prognostic signature in BLCA. (**A**) The 10-fold cross-validation for variable selection in the LASSO model. (**B**) The LASSO coefficient profile of drlncRNAs. DrlncRNAs, disulfidptosis-related long noncoding RNAs.

To develop a risk model incorporating drlncRNAs in BLCA, we randomly assigned 403 BLCA cases to the training and test sets at a 1:1 ratio. Some BLCA cases with clinical data were excluded. The chi-square test indicated that the two groups were comparable in terms of both clinicopathologic and demographic parameters (Table 1). Subsequently, a total of 22 drlncRNAs were selected and validated through Lasso regression to construct this model (Fig. 2A-B). A multivariable Cox regression analysis was conducted to identify nine genes (AC010331.1, LINC-PINT, AC006160.1, LINC00536, AL590428.1, MSC-AS1, AC104785.1, AC243654.1 and AC022364.1) for the development of a prognostic model named “disulfidptosis-score”. Based on the results of the multivariable Cox regression analysis, we constructed the disulfidptosis-score:

$$\begin{array}{l}Risk{\rm{ }}score{\rm{ }} = {\rm{ }}AC010331.1{\rm{ }} \times {\rm{ }}\left( { - 0.22} \right){\rm{ }} + {\rm{ }}LINC - PINT{\rm{ }} \times {\rm{ }}\\\left( { - 1.06} \right){\rm{ }} + {\rm{ }}AC006160.1{\rm{ }} \times {\rm{ }}\left( {1.18} \right){\rm{ }} + {\rm{ }}LINC00536{\rm{ }} \times {\rm{ }}\\\left( {0.39} \right){\rm{ }} + {\rm{ }}AL590428.1{\rm{ }} \times {\rm{ }}\left( {0.53} \right){\rm{ }} + {\rm{ }}MSC - AS1 \times \left( {0.32} \right)\\{\rm{ }} + {\rm{ }}AC104785.1{\rm{ }} \times {\rm{ }}\left( { - 0.26} \right){\rm{ }} + {\rm{ }}\\AC243654.1{\rm{ }} \times {\rm{ }}\left( { - 0.67} \right){\rm{ }} + {\rm{ }}AC022364.1{\rm{ }} \times {\rm{ }}\left( {0.45} \right)\end{array}$$

**Fig. 3:**
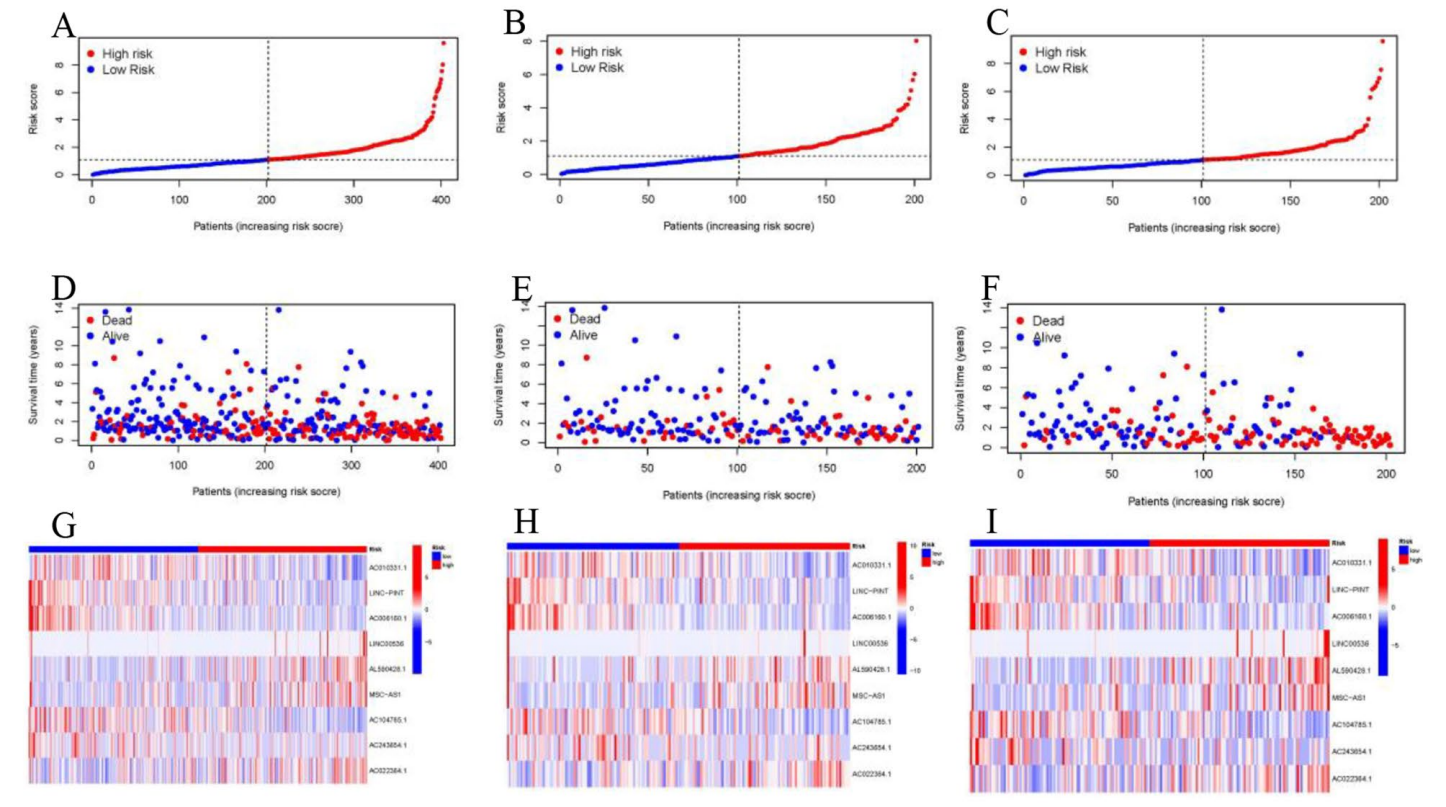
Prognosis capability of the model in the three patient sets. (**A–C**) Distribution of patient with different scores. (**D–F**) Distribution of patient survival time. (**G–I**) The heatmap of nine drlncRNAs expression. DrlncRNAs, disulfidptosis-related long noncoding RNAs.

**Fig. 4:**
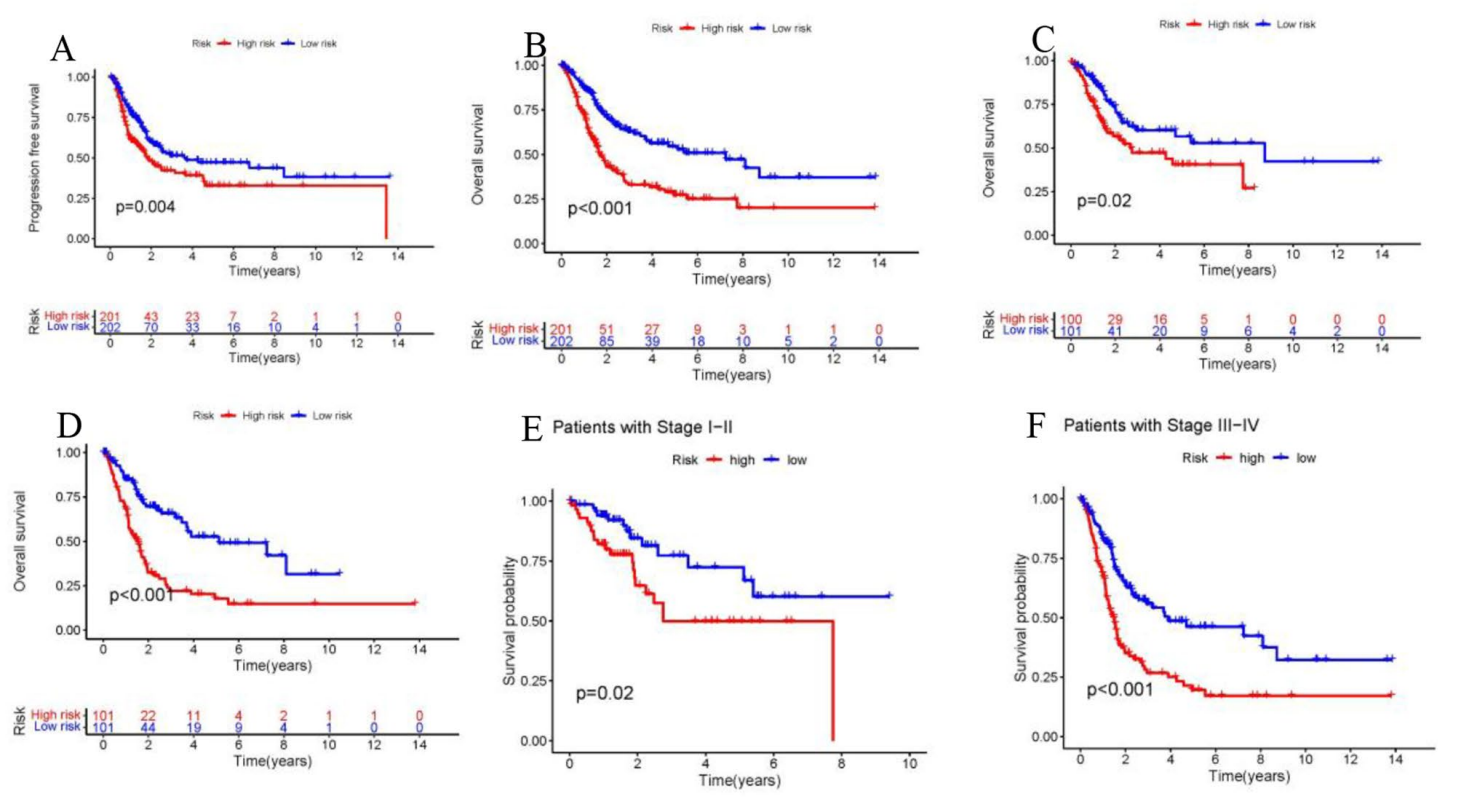
Survival analysis. (**A**) Kaplan-Meier curves of progression-free survival in the entire set. (**B-D**) Comparison of Kaplan-Meier curves between high and low expression groups of entire, test and train set. (**E**, **F**) Kaplan-Meier curves of stratified by clinicopathologic characteristics in the entire set

**Fig. 5:**
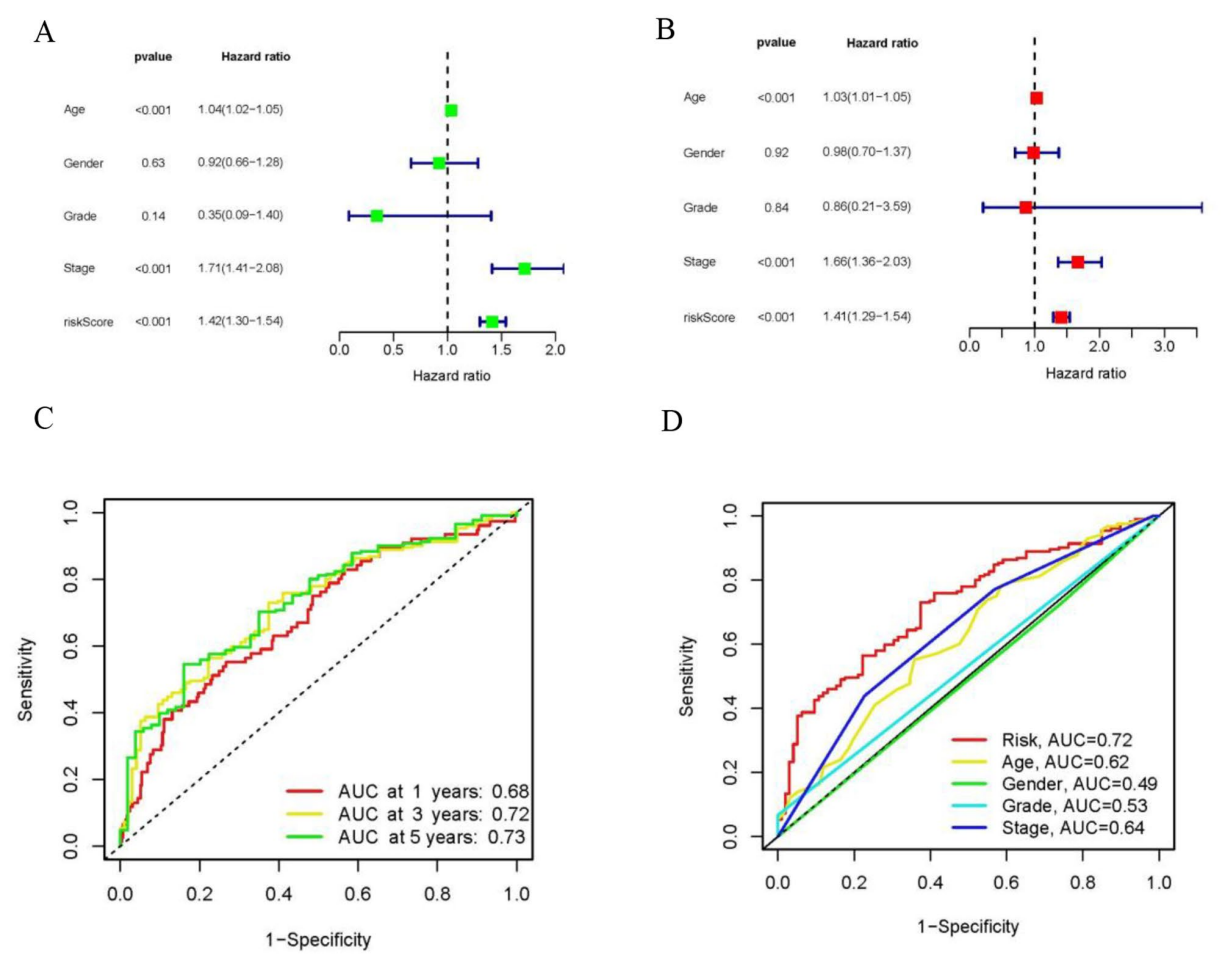
Assessment of the risk model. (**A, B**) Uni- and multi-Cox analyses of clinical factors and risk score with OS. (**C**) The 1-, 3-, and 5-years ROC curves of the entire sets. (**D**) The 3-year ROC curves of risk score and clinical characteristics. OS, overall survival. ROC, receiver operating characteristic

The distribution plot of the disulfidptosis-score revealed a negative correlation between survival times and increasing disulfidptosis-score (Fig. 3A–I). Figure 4A-F demonstrate that low-risk patients have a superior overall survival compared to high-risk patients. To confirm the model’s status as an independent prognostic predictor, both univariable and multivariable Cox regression analyses were conducted. In both univariable and multivariable Cox regression, the HR and 95% CI of risk scores were 1.42 (1.30–1.54) and 1.41 (1.29–1.54), respectively, with *p* < 0.001 (Fig. 5A, B). Age (*p* < 0.001) and stage (*p* < 0.001) were also found to be independent prognostic factors (Fig. 5B). Moreover, the model’s susceptibility and specificity in predicting prognosis were evaluated using time-dependent ROC analysis. The AUC values for overall survival at 1, 3, and 5 years were 0.69, 0.72, and 0.73 respectively (Fig. 5C), and the model’s risk score AUC was 0.72, indicating a stronger predictive power compared to other clinicopathological characteristics (Fig. 5D).

**Table 1:** Clinicopathologic and demographic characteristics of BLCA patients in the training and test cohorts

Covariates	Type	Total	Test	Train	*P*value
Age	<=65	159(39.5%)	84(41.8%)	75(37.1%)	0.4
	> 65	244(60.6%)	117(58.2%)	127(62.9%)	
Gender	FEMALE	106(26.3%)	56(27.9%)	50(24.8%)	0.6
	MALE	297(73.7%)	145(72.1%)	152(75.3%)	
Grade	High Grade	380(94.3%)	187(93.0%)	193(95.5%)	0.5
	Low Grade	20(5.0%)	12(6.0%)	8(4.0%)	
	unknow	3(0.7%)	2(1%)	1(0.5%)	
Stage	Stage I	2(0.5%)	0(0%)	2(1.0%)	0.1
	Stage II	127(31.5%)	71(35.3%)	56(27.7%)	
	Stage III	140(34.7%)	72(35.8%)	68(33.7%)	
	Stage IV	132(32.8%)	56(27.9%)	76(37.6%)	
	unknow	2(0.5%)	2(1.0%)	0(0%)	
T	T1	3(0.8%)	1(0.5%)	2(1.0%)	0.2
	T2	117(29.0%)	67(33.3%)	50(24.8%)	
	T3	193(47.9%)	92(45.8%)	101(50%)	
	T4	57(14.1%)	24(11.9%)	33(16.3%)	
	unknow	33(8.2%)	17(8.5%)	16(7.9%)	
M	M0	194(48.1%)	105(52.2%)	89(44.1%)	0.8
	M1	11(2.7%)	7(3.5%)	4(2.0%)	
	unknow	198(49.2%)	89(44.3%)	109(53.9%)	
N	N0	234(58.1%)	128(63.7%)	106(52.5%)	0.9
	N1	46(11.4%)	17(8.5%)	29(14.4%)	
	N2	75(18.6%)	34(16.9%)	41(20.3%)	
	N3	6(1.5%)	2(1%)	4(1.9%)	
	unknow	42(10.4%)	20(9.9%)	22(10.9%)	

### Establishment of a nomogram scoring system

**Fig. 6:**
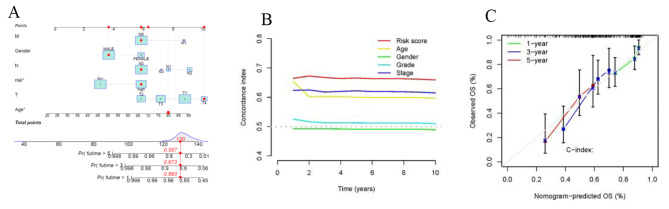
Construction of nomogram. (**A**) Nomogram for predicting overall survival. (**B**)The decision curves. (**C**) The calibration curves for 1-, 3-, and 5-years OS.

Based on the age, gender, risk score, stage, and other clinical features described above, we developed a nomogram that incorporates the disulfidptosis-score and clinicopathological characteristics to predict 1-, 3-, and 5-year OS rates in patients with BLCA (Fig. 6A). We utilized the calibration plots for 1, 3, and 5 years to confirm whether the nomogram corresponded well with predictions (Fig. 6B). The high value of C index indicated that the nomogram has excellent ability to distinguish (Fig. 6C).

### Principal component analysis (PCA) and biological pathways analyses

**Fig. 7:**
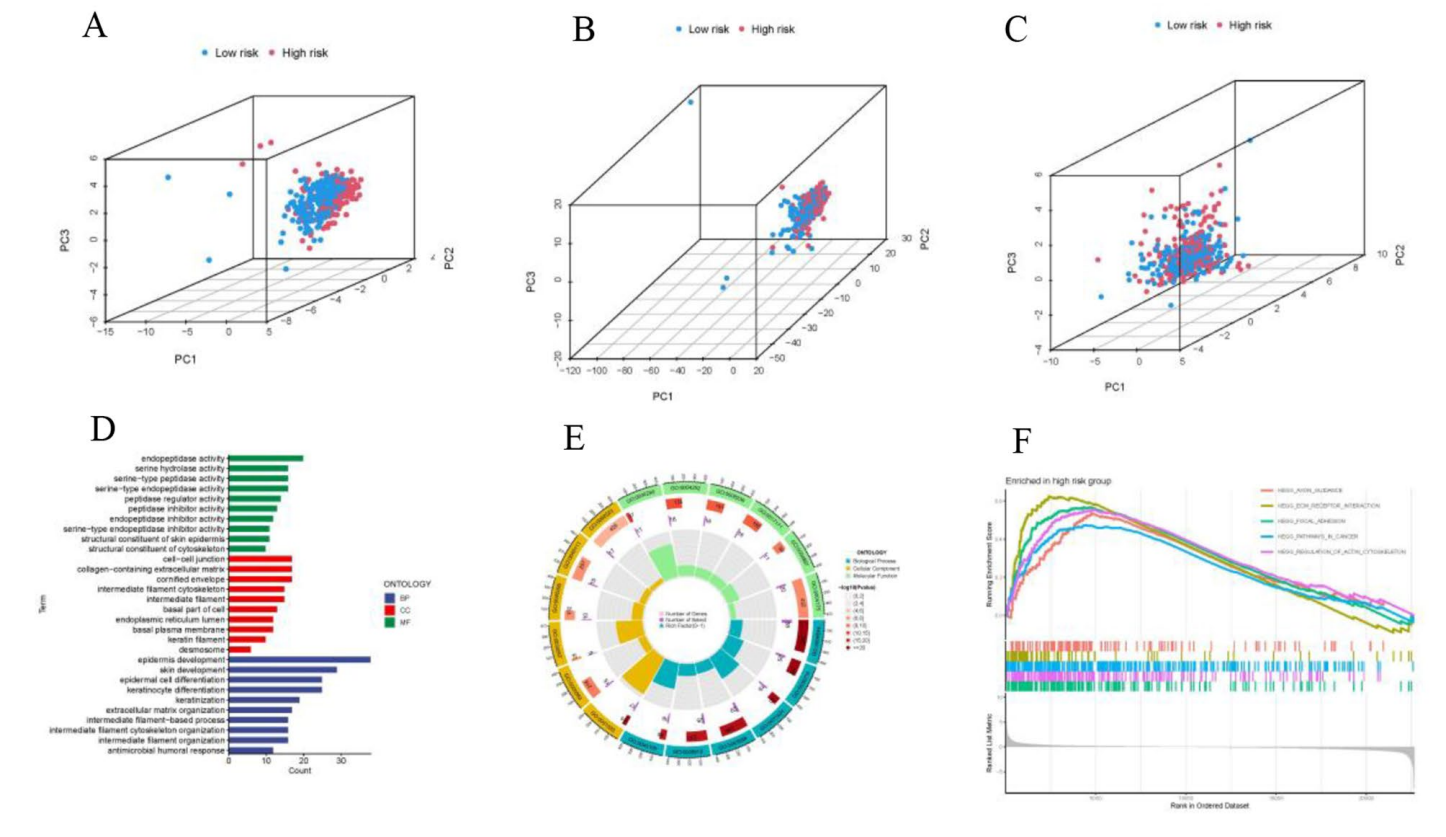
PCA, GO, and GSEA analyses. (**A-C**) 3D scatter plots of risk-LncRNA, disulfidptosis-LncRNA and disulfidptosis-Gene sample distribution. (**D, E**) GO analysis of biological processes, cellular components and molecular functions. (**F**) Results of GSEA analyses

The 3D scatter diagram revealed distinct aggregation features of PCA for the low-risk and high-risk groups (Fig. 7A-C). Gene ontology (GO) analysis indicated related biological processes included epidermis development, skin development, cell − cell junction, cornified envelope, collagen − containing extracellular matrix, endopeptidase activity, and serine hydrolase activity (Fig. 7D,E). To explore the functional disparities in gene expression between high- and low-risk groups, we conducted a functional enrichment analysis utilizing Gene Set Enrichment Analysis (GSEA) software. GSEA identified genes involved in Axon guidance, Extracellular matrix (ECM) receptor interaction, Focal adhesion, Pathways in cancer, and regulation of action cytoskeleton (Fig. 7F).

### Correlation analysis between risk scores and gene mutations

**Fig. 8:**
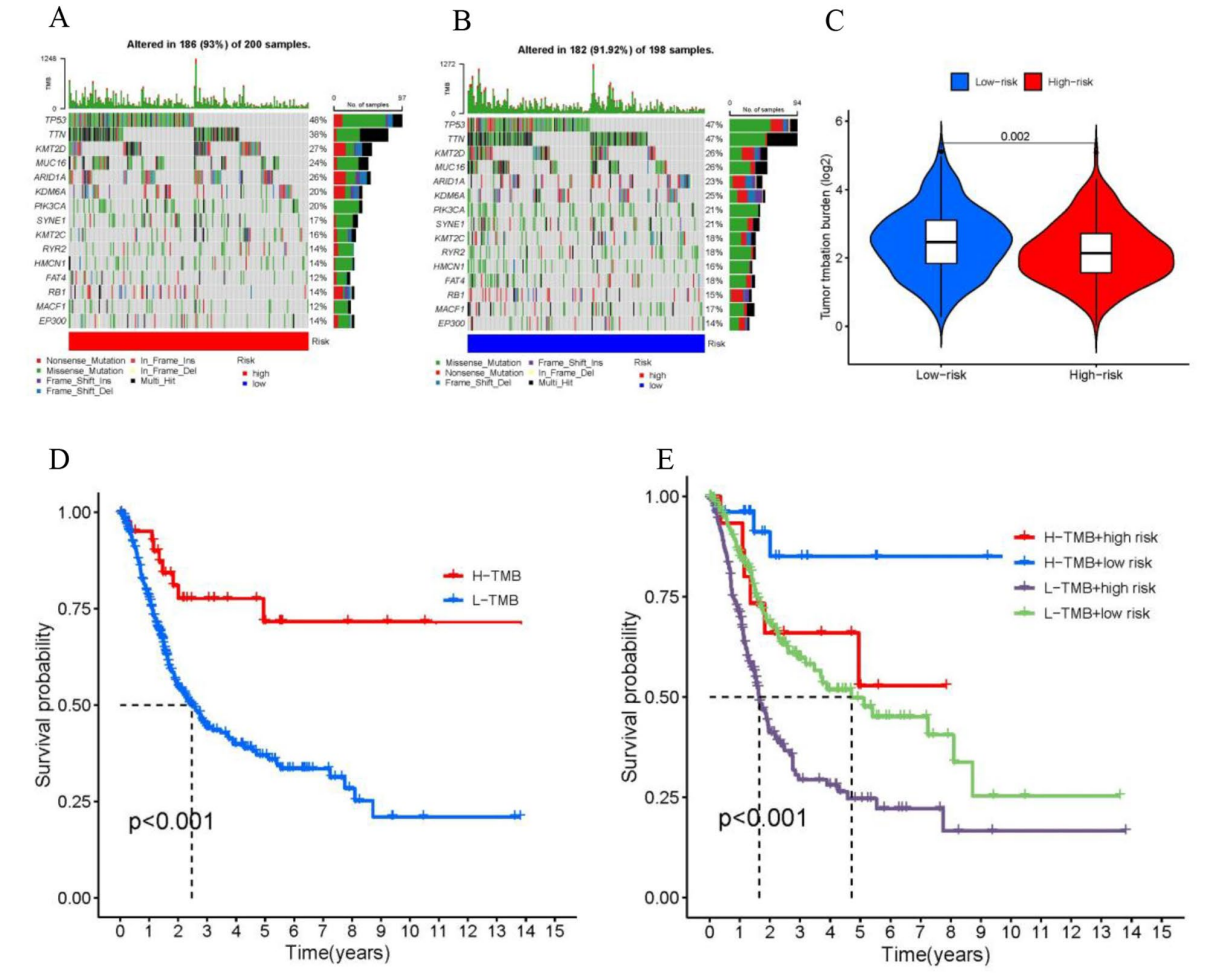
Tumor mutation burden (TMB). (**A, B**) Waterfall plot of top 15 mutation genes in the high-risk and low-risk group in BLCA. (**C**) There were significantly higher TMB in the low-risk group compared to the high-risk group. (**D**) Kaplan-Meier curves show similar patient survival between the high- and the low-TMB groups. (**E**) Kaplan-Meier curves show different patient survival among the four groups

Somatic mutations were compared between the two groups. We have identified the top 15 genes with the highest mutation rates in both high-risk (Fig. 8A) and low-risk groups (Fig. 8B). The mutation rates of TP53, TTN, and KMT2D were not only higher than 25% in both groups but also the most common mutations observed. Furthermore, we investigated the association between risk score and tumor mutational burden (TMB). We found that TMB was significantly greater in the low-risk group compared to the high-risk group (Fig. 8C). Patients with higher scores and lower TMB exhibited the poorest prognosis among the four groups (Fig. 8D, E).

### TIDE, immune functions and prediction of clinical treatment response

**Fig. 9:**
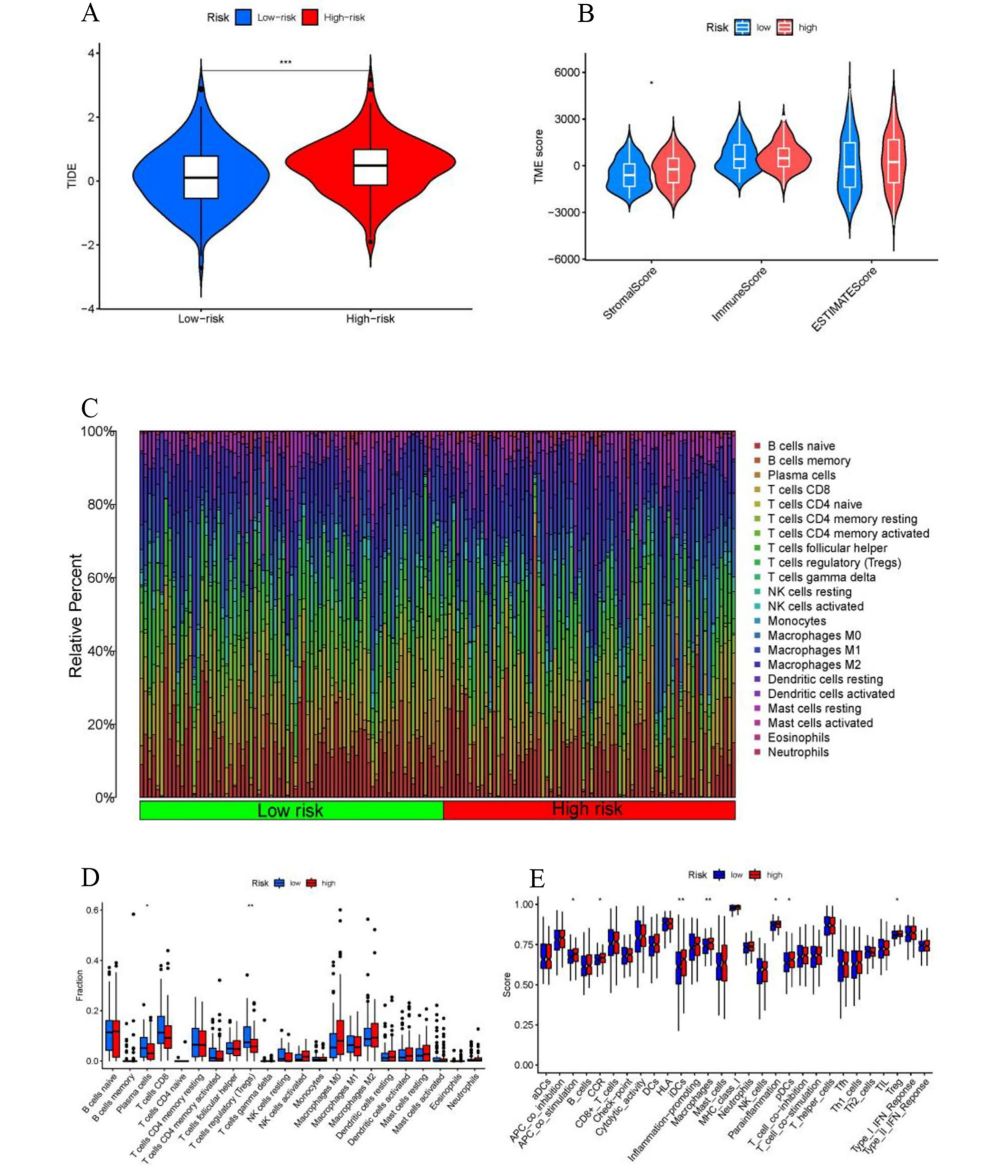
Immune functions and prediction of clinical treatment response. (**A**) TIDE scores. (**B**) Differential analysis of tumor microenvironment. (**C**) Composition of immune cells in two disulfidptosis-score subgroup. (**D**) The Relative immune infiltration score of 22 immune cells between low- and high-risk groups. (**E**) Analysis of immune function differences. TIDE, tumor immune dysfunction and exclusion

The TIDE scores exhibited a significant increase in the high-risk subgroup as compared to the low-risk subgroup. This indicated that disulfidptosis-score has the potential to assess the responsiveness of BLCA patients to immune checkpoint blockade therapy (Fig. 9A). Differential analysis of tumor microenvironment indicated differences in stromal-score between high- and low-risk groups (Fig. 9B). We also examined the immune cell composition among different risk groups (Fig. 9C) in the TCGA database of BLCA samples. The results indicated that the low-risk subgroup of patients had significantly higher proportions of plasma cells and regulatory T cells (*p* < 0.05) (Fig. 9D). Analysis revealed differential expression of immune function in APC co-stimulation, CCR, iDCs, macrophages, parainflammation, pDCs and Treg between the low-risk and high-risk groups (Fig. 9E). In addition, research on the susceptibility of different patient groups to antitumor drugs indicates that low-risk groups exhibit greater sensitivity to certain drugs such as Doramapimod, Elephantin, and Nilotinib, while high-risk groups are more responsive to Dasatinib, Sapitinib, and Staurosporine (Supplementary Figure S2).

## Discussion

BLCA is a highly aggressive malignancy with a dismal prognosis [13]. Treatment options for advanced BLCA have expanded to immune checkpoint inhibitors, targeted therapies, and Antibody–Drug Conjugates [2, 14]. Despite the rapid development of treatment options, choices for treating BLCA have remained limited until now, resulting in a discouraging prognosis for advanced cases under primary treatment. Disulfidptosis is a unique form of cell death that differs from apoptosis, cuproptosis, ferroptosis, pyroptosis and necrosis [7, 8, 15]. Investigating disulfidptosis-related genes in cancer can aid in understanding the mechanisms of tumor development. Therefore, this study identified a set of 10 BLCA-related disulfidptosis genes and developed a disulfidptosis-risk score to predict prognosis and provide guidelines for individualized clinical strategies for BLCA.

During our research, we explored the role of drlncRNA in BLCA and identified several lncRNAs that have prognostic value. Using Cox regression and Lasso regression, we developed a prognostic model consisting of nine lncRNAs (AC010331.1, LINC-PINT, AC006160.1, LINC00536, AL590428.1, MSC-AS1, AC104785.1, AC243654.1, and AC022364.1). LINC-PINT is a promising prognostic marker for bladder cancer, and its upregulation can suppress the proliferation, invasion, and migration of bladder cancer cells by targeting miR-155-5p [16]. The lncRNA AC006160.1 may serve as a protective factor for the progression of bladder cancer [17]. Li et al. found that LINC00536 promoted BC progression by modulating the Wnt3a/β-Catenin signaling [18]. Hypermethylated lncRNA MSC-AS1 has the potential to be prognostic biomarkers for bladder cancer [19]. In bladder cancer, AC104785.1, AL590428.1, and AC010331.1 were risk factors for BLCA patients [20, 21]. At present, there is no profound study for AC243654.1 and AC022364.1.

To enhance the precision of prognostic prediction, we have developed and validated a nomogram by carefully selecting various factors including disulfidptosis-score, age, gender, grade, TNM stage and pathological stage. The results of the independent prognostic analysis showed that age, disulfidptosis-score, and pathological stage were significantly associated with the prognosis of BLCA. Additionally, we identified the disulfidptosis-score as an independent prognostic factor.

Numerous studies on various tumors have shown that patients with high TMB often have higher survival rates [22]. Similarly, we observed that the low-risk group of disulfidptosis-score exhibited higher TMB levels. Upon comparing somatic mutations between the two groups, we observed a higher frequency of mutations in the low-risk group. The waterfall plot showed that TP53 and TTN mutations were more frequent in both groups of BLCA patients. TP53 gene mutation is one of the most common mutations in human BLCA and has been implicated in the progression and prognosis of this disease [23]. Li et al. found that TTN-AS1 serves as an oncogene by activating ATF2 in BLCA [24].

To investigate the significance of immune cell infiltration in BLCA among different risk groups, we used CIBERSORT to analyze the relative proportion of immune cells in each BLCA specimen for our study [25]. The presence of immune infiltration and immune checkpoints in low-risk groups suggests a higher number of highly infiltrated Tregs, which are more active in promoting tumor immune evasion [26]. Therefore, the low-risk group may be more effective for immunotherapy. According to the evidence, we believe that the disulfidptosis-score has the potential to reflect both immune cell infiltration and the prognostic significance of various immune cell types.

According to the clinical trial, literature has demonstrated that immunotherapy produces remarkable outcomes in BLCA patients prior to achieving disease control through standard chemotherapy [27–29]. We aimed to investigate whether the combination of chemotherapy and immune therapy in BLCA patients had superior efficacy for further research. Therefore, we assessed drug sensitivity among two different risk groups. Additionally, we identified a strong correlation between high-risk group and drug sensitivity, particularly with regards to Dasatinib, Sapitinib, and Staurosporine. Ultimately, our findings suggested that the disulfidptosis-score can serve as both a prognostic tool and guide for individualized treatment.

The present study has limitations and deficiencies that need to be addressed. Firstly, our investigation was solely based on the TCGA dataset without any in vitro or in vivo verification of the results obtained. Further exploration is required to fully understand the biological functions involved. Secondly, the study reported an interval validation only. Experimental validation is required to confirm the potential molecular mechanism of drlncRNAs in BLCA. Lastly, due to a lack of clinical follow-up data, we were unable to demonstrate the value of our prognostic model.

## Conclusion

Our research demonstrated that BLCA patients based on their disulfidptosis-score can help differentiate clinicopathological features, immune infiltration, and clinical prognosis. Furthermore, this study highlights the prognostic value of the disulfidptosis-score and offers insights for personalized treatment strategies involving immunotherapy and chemotherapy. However, further research is needed to explore the biological mechanisms that underlie interactions among these model genes.

### Electronic supplementary material

Below is the link to the electronic supplementary material.

Supplementary Material 1

Supplementary Material 2

## Data Availability

The datasets analysed during the current study are available in the TCGA database (https://portal.gdc.cancer.gov/). The original contributions presented in the study are included in the article/Supplementary Material, further inquiries can be directed to the corresponding authors.

## Abbreviations

DRGs: Disulfidptosis-related genes
DrlncRNAs: Disulfidptosis-related long noncoding RNAs
BLCA: Bladder urothelial carcinoma
TME: Tumor microenvironment
TCGA: The Cancer Genome Atlas
GO: Gene ontology
PCA: Principal component analysis
IC50: Half-maximal inhibitory concentration prediction
PFS: Progression-Free-Survival
C-index: Concordance index
ROC: Receiver operating characteristic
DEGs: Differentially expressed genes
GSEA: Gene Set Enrichment Analysis
TMB: Tumor mutation burden
TIDE: Tumor immune dysfunction and exclusion
AUC: Area under the curve
