# Supplementary material for: Preliminary findings on the development of a predictive model for BLCA based on disulfidptosis-associated IncRNAs signature
Source: BMC Urol. 2024 Mar 26;24:69. doi: 10.1186/s12894-024-01454-3 (PMC10964663; doi:10.1186/s12894-024-01454-3)
Supplement: Supplementary file 1 — Supplementary Material 1 [file 12894_2024_1454_MOESM1_ESM.docx]

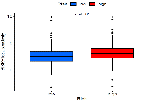

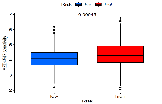

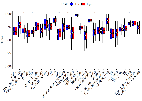

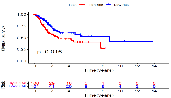

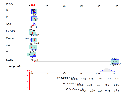

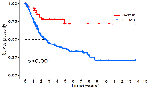

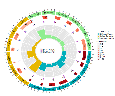

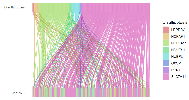

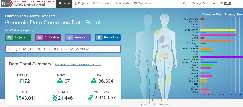

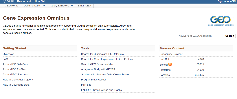


Drug sensitivity analysis

Correlation of risk scores with immunotherapy response

Validation of the risk score model

The nomogram based on risk score in BLCA

Data collection

Correlation of drlncRNAs with TME, GO and KEGG

Genetic expression of drlncRNAs in BLCA

Supplementary Figure S1: The flowchart of the study. drlncRNAs: disulfidptosis-related long noncoding RNAs.
