# Supplementary material for: Preliminary findings on the development of a predictive model for BLCA based on disulfidptosis-associated IncRNAs signature
Source: BMC Urol. 2024 Mar 26;24:69. doi: 10.1186/s12894-024-01454-3 (PMC10964663; doi:10.1186/s12894-024-01454-3)
Supplement: Supplementary file 2 — Supplementary Material 2 [file 12894_2024_1454_MOESM2_ESM.docx]

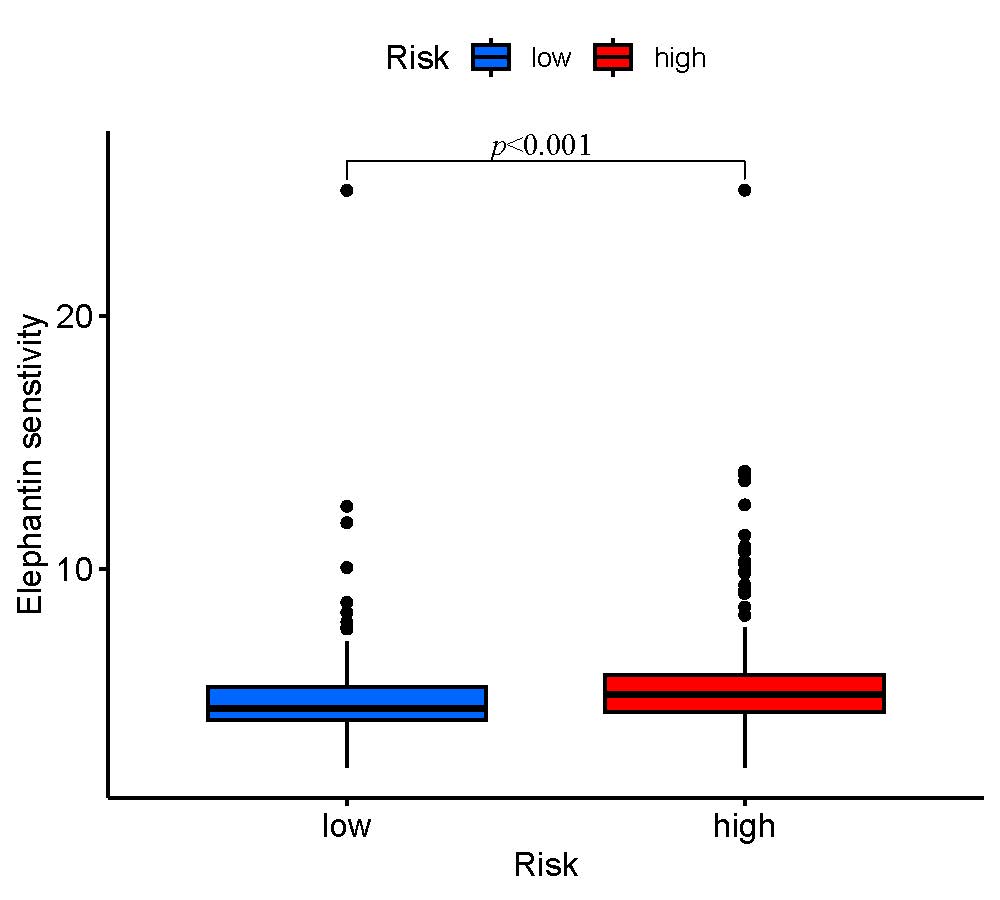

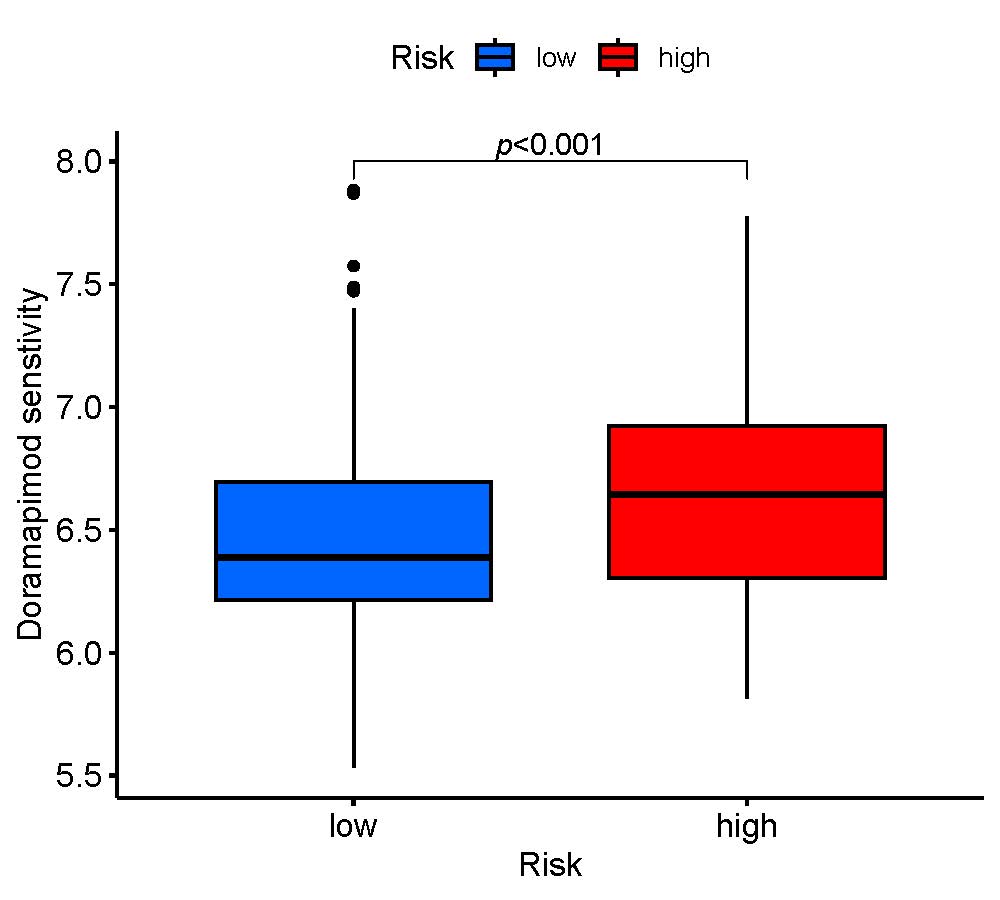

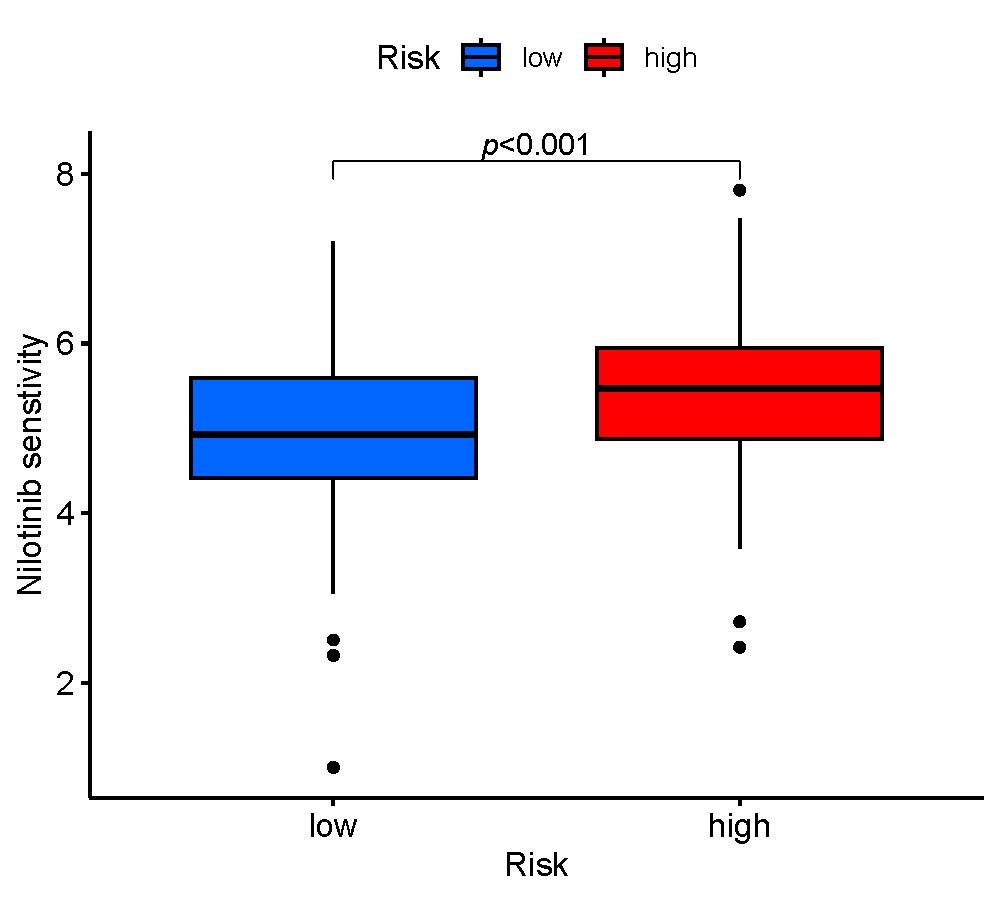


C

B

A


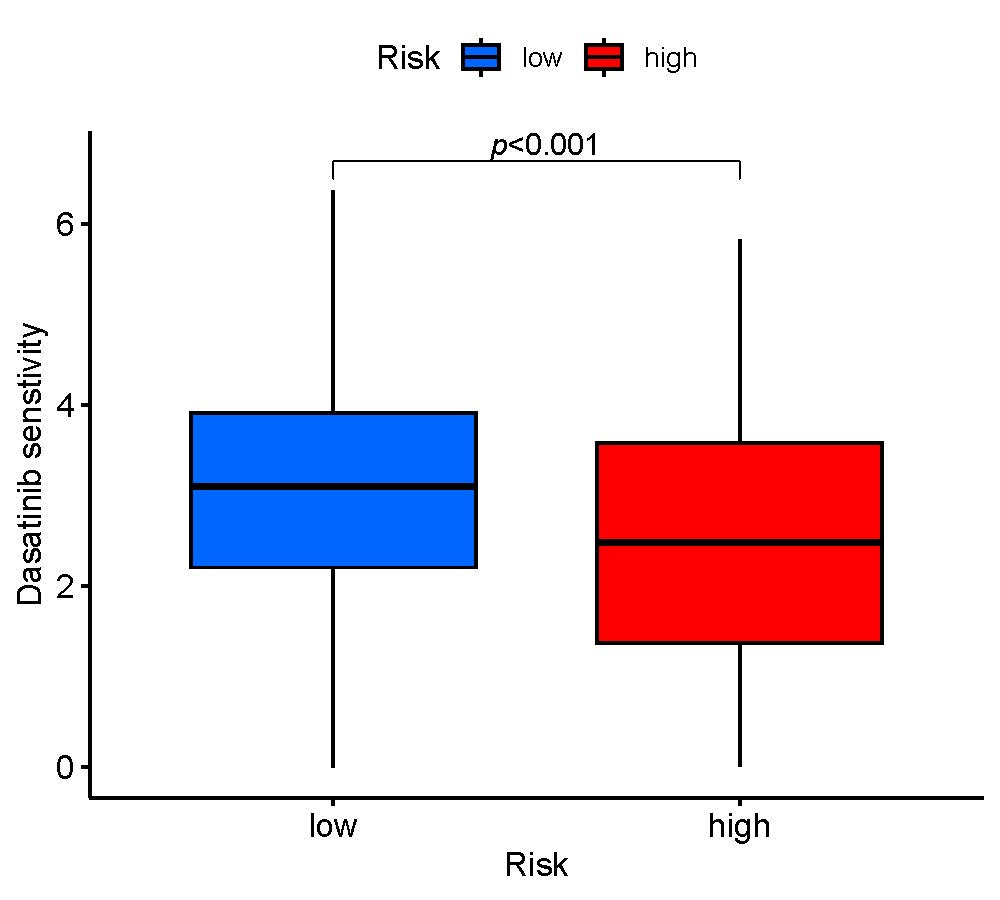


F

E

D


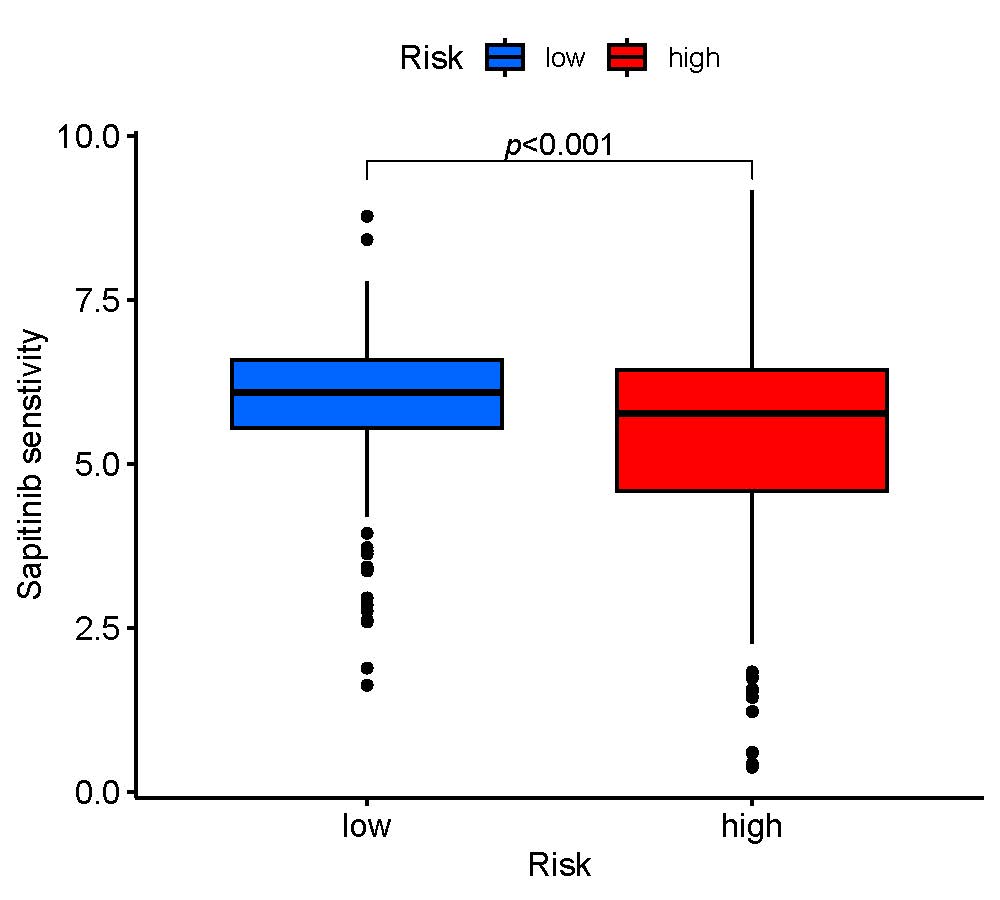

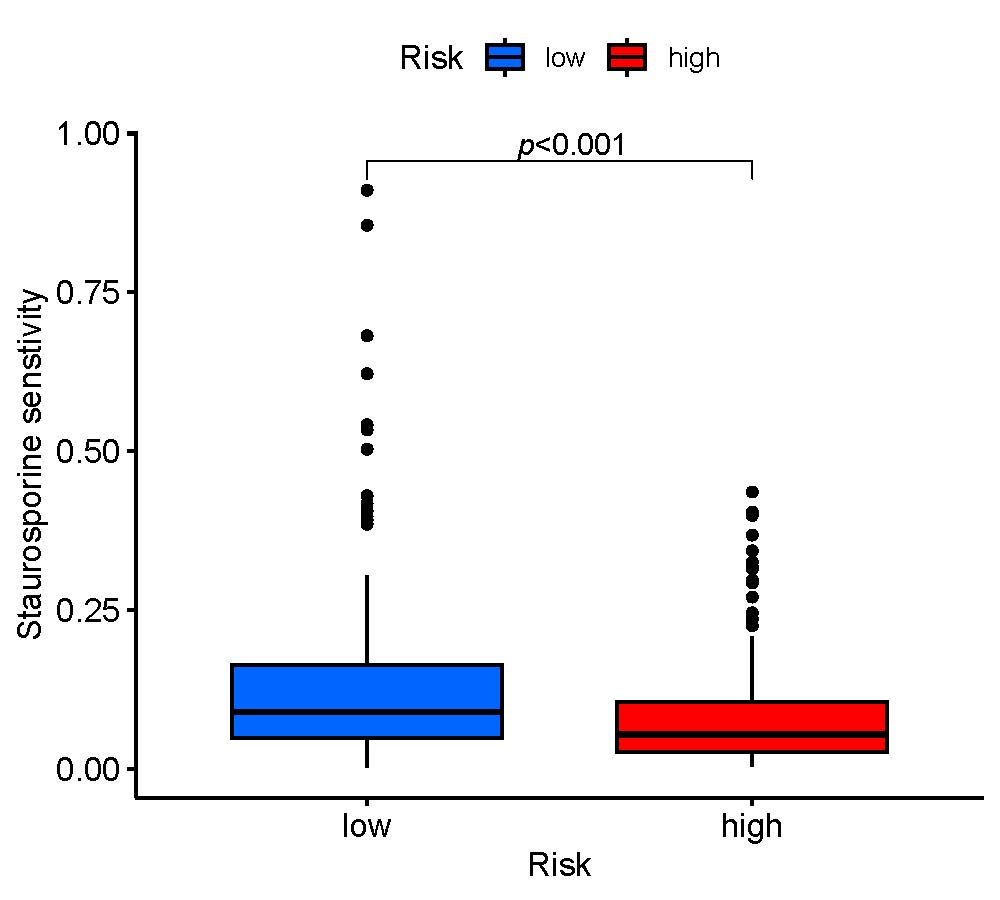


Supplementary Figure S2: Relationships between disulfidptosis-score and medicine sensitivity. Lower IC50 of indicated chemo-therapeutics drugs in low (A-C) and high (D-F) disulfidptosis-score group, respectively. IC50, half-maximal inhibitory concentration prediction.
